# GENDER DIFFERENCES ON THE EFFECT OF RECIPROCITY ON NEGLECT AND ABUSE BY DEMENTIA CAREGIVERS

**DOI:** 10.1093/geroni/igae098.0500

**Published:** 2024-12-31

**Authors:** Vicki Winstead, Carolyn Pickering, Wesley Browning, Jessica Hernandez, Mustafa Yildiz

**Affiliations:** The University of Texas Health Science Center, Houston, Texas, United States; The University of Texas Health Houston, Houston, Texas, United States; The University of Texas Health Science Center Houston, Houston, Texas, United States; The University of Texas Health Houston, Houston, Texas, United States; The University of Texas Health Houston, Houston, Texas, United States

## Abstract

Reciprocity, a balanced exchange in interactions, is a crucial element of most social exchanges and impacts caregiving outcomes. This paper aims to investigate whether reciprocity plays a protective role against elder abuse and neglect and to explore potential gender variations associated with this relationship. A sample of 453 family caregivers reported daily perceptions of reciprocity in a micro-longitudinal study over 21 days. Perceived reciprocity was measured by the participant’s level of satisfaction with the amount of affection/appreciation shown by their relative with dementia in the previous 24 hours. Generalized linear mixed models examined the effect of perceived reciprocity on the odds of the caregiver engaging in psychologically aggressive, physically aggressive, and neglectful behaviors that day. Perceived reciprocity was associated with lowered daily odds of neglect (OR= 0.76 p= 0.006) and psychological abuse (OR = 0.6 p< 0.001). We calculated an interaction term of the effects of gender and perceived reciprocity on abuse/neglect to assess gender differences. Increased reciprocity reduced the odds of neglect for both males and females, but the reduction in odds was greater for males (p=0.041). Results suggest that receiving attention and affection for caregiving may act as a protective factor, particularly for male caregivers. While female caregivers might face socially constructed role expectations, males, often taking on caregiving out of obligation, may benefit from reciprocal interactions regarding reduced likelihood of neglecting the care recipient. These insights contribute to a nuanced understanding of gender dynamics in caregiving and can inform interventions to prevent elder abuse and neglect.

